# Supplementary figures and images for: A semantics, energy-based approach to automate biomodel composition
Source: PLoS One. 2022 Jun 3;17(6):e0269497. doi: 10.1371/journal.pone.0269497 (PMC9165793; doi:10.1371/journal.pone.0269497)

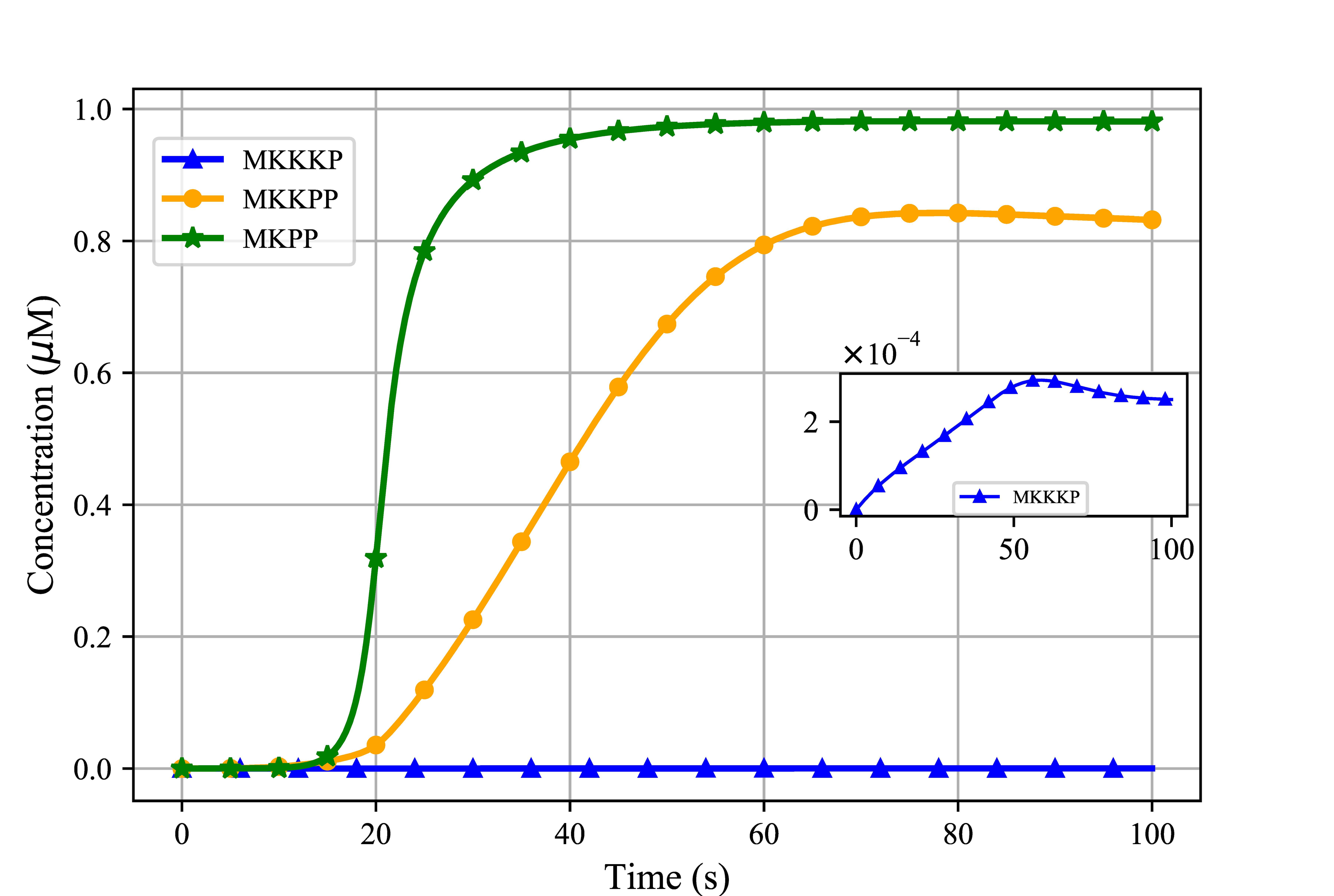

Supplement: S1 Fig — For an input kinase of Ras = 3 × 10^−5^ (μM), the concentration changes of the activated kinases (MKKKP, MKKPP, and MKPP) show the signal is amplified through each layer. (TIF) [file pone.0269497.s001.tif]

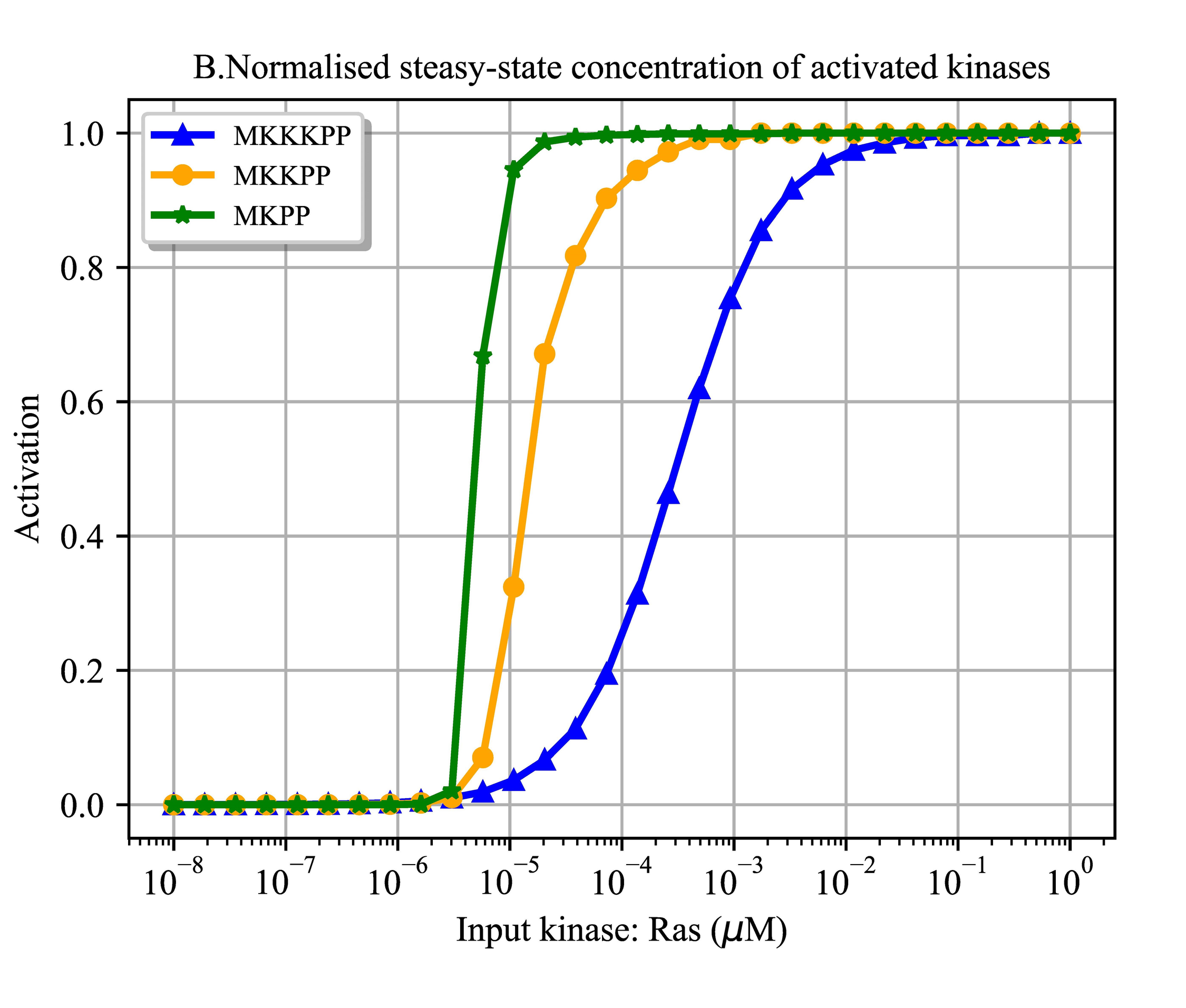

Supplement: S2 Fig — (TIF) [file pone.0269497.s002.tif]
