# Supplementary material for: A semantics, energy-based approach to automate biomodel composition
Source: PLoS One. 2022 Jun 3;17(6):e0269497. doi: 10.1371/journal.pone.0269497 (PMC9165793; doi:10.1371/journal.pone.0269497)
Supplement: S1 Table — Steps 4, 8, and 16 are irreversible reactions, which are approximated by mass action kinetics. κi(i ∈ {Step}) in the reaction rate equations represent the reaction rate constants, Kx (x ∈ {Reactants, Products}) is the thermodynamic constant of each species, and qx (x ∈ {Reactants, Products}) is the concentration amount of each species. (PDF) [file pone.0269497.s003.pdf]

S1 Table. Reactant(s) and product(s) of each step in EGFR pathway and the reaction rate equations.

Steps 4, 8, and 16 are irreversible reactions, which are approximated by mass action kinetics. κ_i ($i \in \{Step\}$) in the reaction rate equations represent the reaction rate constants, K_x ($x \in \{Reactant(s), Product(s)\}$) is the thermodynamic constant of each species, and q_x ($x \in \{Reactant(s), Product(s)\}$) is the concentration amount of each species.

Step	Reactant(s)		Product(s)			Reaction rate equation (law of mass action)
1	EGF	$EGFR$	$EGF{:}EGFR$ $complex$	---	---	$\kappa_1(K_{EGFR}.q_{EGFR}.K_{EGF}.q_{EGF} - K_{(EGF:EGFR)}.q_{(EGF:EGFR)})$
2	$EGF{:}EGFR$ $complex$	$EGF{:}EGFR$ $complex$	R_2	---	---	$\kappa_2({K^2}_{(EGF:EGFR)}.{q^2}_{(EGF:EGFR)} - K_{R_2}.q_{R_2})$
3	R_2	---	RP	---	---	$\kappa_3(K_{R_2}.q_{R_2} - K_{RP}.q_{RP})$
4	RP	ATP	R_2	ADP	P	$\kappa_4(K_{RP}.q_{RP}.K_{ATP}.q_{ATP} - K_{R_2}.q_{R_2}.K_{ADP}.q_{ADP}.K_P.q_P)$
5	RP	PLC_γ	RPL	---	---	$\kappa_5(K_{RP}.q_{RP}.K_{PLC_\gamma}.q_{PLC_\gamma} - K_{RPL}.q_{RPL})$
6	RPL	---	$RPLP$	---	---	$\kappa_6(K_{RPL}.q_{RPL} - K_{RPLP}.q_{RPLP})$
7	$RPLP$	---	RP	$PLC_\gamma P$	---	$\kappa_7(K_{RPLP}.q_{RPLP} - K_{RP}.q_{RP}.K_{PLC_\gamma P}.q_{PLC_\gamma P})$
8	$PLC_\gamma P$	ATP	PLC_γ	ADP	P	$\kappa_8(K_{PLC_\gamma P}.q_{PLC_\gamma P}.K_{ATP}.q_{ATP} - K_{PLC_\gamma}.q_{PLC_\gamma}.K_{ADP}.q_{ADP}.K_P.q_P)$
9	RP	Grb	RG	---	---	$\kappa_9(K_{RP}.q_{RP}.K_{Grb}.q_{Grb} - K_{RG}.q_{RG})$
10	RG	SOS	RGS	---	---	$\kappa_{10}(K_{RG}.q_{RG}.K_{SOS}.q_{SOS} - K_{RGS}.q_{RGS})$
11	RGS	---	RP	GS	---	$\kappa_{11}(K_{RGS}.q_{RGS} - K_{RP}.q_{RP}.K_{GS}.q_{GS})$
12	GS	---	Grb	SOS	---	$\kappa_{12}(K_{GS}.q_{GS} - K_{Grb}.q_{Grb}.K_{SOS}.q_{SOS})$
13	RP	Shc	RSh	---	---	$\kappa_{13}(K_{RP}.q_{RP}.K_{Shc}.q_{Shc} - K_{RSh}.q_{RSh})$
14	RSh	---	$RShP$	---	---	$\kappa_{14}(K_{RSh}.q_{RSh} - K_{RShP}.q_{RShP})$
15	$RShP$	---	RP	ShP	---	$\kappa_{15}(K_{RShP}.q_{RShP} - K_{RP}.q_{RP}.K_{ShP}.q_{ShP})$
16	ShP	ATP	Shc	ADP	P	$\kappa_{16}(K_{ShP}.q_{ShP}.K_{ATP}.q_{ATP} - K_{Shc}.q_{Shc}.K_{ADP}.q_{ADP}.K_P.q_P)$
17	$RShP$	Grb	$RShG$	---	---	$\kappa_{17}(K_{RShP}.q_{RShP}.K_{Grb}.q_{Grb} - K_{RShG}.q_{RShG})$
18	$RShG$	---	RP	ShG	---	$\kappa_{18}(K_{RShG}.q_{RShG} - K_{RP}.q_{RP}.K_{ShG}.q_{ShG})$
19	$RShG$	SOS	$RShGS$	---	---	$\kappa_{19}(K_{RShG}.q_{RShG}.K_{SOS}.q_{SOS} - K_{RShGS}.q_{RShGS})$
20	$RShGS$	---	$ShGS$	RP	---	$\kappa_{20}(K_{RShGS}.q_{RShGS} - K_{ShGS}.q_{ShGS}.K_{RP}.q_{RP})$
21	ShP	Grb	ShG	---	---	$\kappa_{21}(K_{ShP}.q_{ShP}.K_{Grb}.q_{Grb} - K_{ShG}.q_{ShG})$
22	ShG	SOS	$ShGS$	---	---	$\kappa_{22}(K_{ShG}.q_{ShG}.K_{SOS}.q_{SOS} - K_{ShGS}.q_{ShGS})$
23	$ShGS$	---	ShP	GS	---	$\kappa_{23}(K_{ShGS}.q_{ShGS} - K_{ShP}.q_{ShP}.K_{GS}.q_{GS})$
24	$RShP$	GS	$RShGS$	---	---	$\kappa_{24}(K_{RShP}.q_{RShP}.K_{GS}.q_{GS} - K_{RShGS}.q_{RShGS})$
25	$PLC_\gamma P$	---	$PLC_\gamma I$	---	---	$\kappa_{25}(K_{PLC_\gamma P}.q_{PLC_\gamma P} - K_{PLC_\gamma I}.q_{PLC_\gamma I})$
