# Supplementary material for: A semantics, energy-based approach to automate biomodel composition
Source: PLoS One. 2022 Jun 3;17(6):e0269497. doi: 10.1371/journal.pone.0269497 (PMC9165793; doi:10.1371/journal.pone.0269497)
Supplement: S2 Table — (PDF) [file pone.0269497.s004.pdf]

S2 Table. Kinetic and bond graph parameters of the species in the EGFR pathway model.

Species	Kholodenko et al.	Bond graph version	
	q (nM)	K (nM^{-1})	q (nM)
EGF	680	0.00345	680
$EGFR$	100	0.00345	100
EGF: $EGFR$ $complex$	0	0.00023	0
R_2	0	5.72797	0
RP	0	1.52491	0
RPL	0	7.00286	0
$RPLP$	0	932.16266	0
PLC_γ	105	1.37768	105
$PLC_\gamma P$	0	12.22572	0
$PLC_\gamma I$	0	0.36677	0
RG	0	0.07026	0
RGS	0	0.07092	0
Grb	85	0.00276	85
SOS	34	0.16826	34
GS	0	0.00697	0
RSh	0	5.79765	0
$RShP$	0	154.34709	0
ShP	0	0.28005	0
ShG	0	0.02584	0
Shc	150	5.70290	150
$RShG$	0	14.23610	0
$ShGS$	0	0.00856	0
$RShGS$	0	5.55987	0
ATP	--	266223.39715	10^3
ADP	--	10^{-3}	10^3
P	--	10^{-3}	10^3
