# Supplementary material for: A semantics, energy-based approach to automate biomodel composition
Source: PLoS One. 2022 Jun 3;17(6):e0269497. doi: 10.1371/journal.pone.0269497 (PMC9165793; doi:10.1371/journal.pone.0269497)
Supplement: S3 Table — (PDF) [file pone.0269497.s005.pdf]

S3 Table. Kinetic and bond graph parameters of the reactions in the EGFR pathway model.

Reactions	Kholodenko et al.		Bond graph version
	k^+	k^-	κ (nmol.s^{-1})
$\boldsymbol{Re}:\boldsymbol{\kappa 1}$	0.003	0.06	250.69800
$\boldsymbol{Re}:\boldsymbol{\kappa 2}$	0.01	0.1	174581.91080
$\boldsymbol{Re}:\boldsymbol{\kappa 3}$	1	0.01	0.00655
$\boldsymbol{Re}:\boldsymbol{\kappa 5}$	0.06	0.2	2855.97575
$\boldsymbol{Re}:\boldsymbol{\kappa 6}$	1	0.05	5.36387
$\boldsymbol{Re}:\boldsymbol{\kappa 7}$	0.3	0.006	0.00032
$\boldsymbol{Re}:\boldsymbol{\kappa 9}$	0.003	0.05	0.711571
$\boldsymbol{Re}:\boldsymbol{\kappa 10}$	0.01	0.06	0.84593
$\boldsymbol{Re}:\boldsymbol{\kappa 11}$	0.03	4.5e-3	0.42304
$\boldsymbol{Re}:\boldsymbol{\kappa 12}$	1.5e-3	1e-4	0.21499
$\boldsymbol{Re}:\boldsymbol{\kappa 13}$	0.09	0.6	10349.01515
$\boldsymbol{Re}:\boldsymbol{\kappa 14}$	6	0.06	0.00038
$\boldsymbol{Re}:\boldsymbol{\kappa 15}$	0.3	9e-4	0.00202
$\boldsymbol{Re}:\boldsymbol{\kappa 17}$	0.003	0.1	0.00702
$\boldsymbol{Re}:\boldsymbol{\kappa 18}$	0.3	9e-4	0.02193
$\boldsymbol{Re}:\boldsymbol{\kappa 19}$	0.01	2.14e-2	0.0040
$\boldsymbol{Re}:\boldsymbol{\kappa 20}$	0.12	2.4e-4	0.01991
$\boldsymbol{Re}:\boldsymbol{\kappa 21}$	0.003	0.1	3.87197
$\boldsymbol{Re}:\boldsymbol{\kappa 22}$	0.03	0.064	7.17844
$\boldsymbol{Re}:\boldsymbol{\kappa 23}$	0.1	0.021	11.2010
$\boldsymbol{Re}:\boldsymbol{\kappa 24}$	0.009	4.29e-2	0.00803
$\boldsymbol{Re}:\boldsymbol{\kappa 25}$	1	0.03	0.08179
	K_m (nmolar)	V_m (nmolar.s^{-1})	
$\boldsymbol{Re}:\boldsymbol{\kappa 4}$	50	450	5.52664
$\boldsymbol{Re}:\boldsymbol{\kappa 8}$	100	1	0.00078
$\boldsymbol{Re}:\boldsymbol{\kappa 16}$	340	1.7	0.014539
