# Supplementary material for: A semantics, energy-based approach to automate biomodel composition
Source: PLoS One. 2022 Jun 3;17(6):e0269497. doi: 10.1371/journal.pone.0269497 (PMC9165793; doi:10.1371/journal.pone.0269497)
Supplement: S4 Table — Steps 2 and 4 are irreversible reactions, which are approximated by mass action kinetics. κi(i ∈ {Step}) in the reaction rate equations represent the reaction rate constants, Kx (x ∈ {Reactants, Products}) is the thermodynamic constant of each species, and qx (x ∈ {Reactants, Products}) is the concentration amount of each species. (PDF) [file pone.0269497.s006.pdf]

S4 Table. Reactant(s) and product(s) of each step in the Ras activation intermediate pathway and the reaction rate equations.

Steps 2 and 4 are irreversible mass action reactions, which are approximated by reversible mass action kinetics. κ_i ($i \in \{Step\}$) in the reaction rate equations represent the reaction rate constants, K_x ($x \in \{Reactant(s), Product(s)\}$) is the thermodynamic constant of each species, and q_x ($x \in \{Reactant(s), Product(s)\}$) is the concentration amount of each species.

Step	Reactant(s)		Product(s)		Reaction rate equation (law of mass action)
**1**	$RasGDP$	$RShcGS$	$RasShcGS$	---	$\kappa_1(K_{RasGDP}\cdot q_{RasGDP}\cdot K_{RShcGS}\cdot q_{RShcGS} - K_{RasShGS}\cdot q_{RasShcGS})$
**2**	$RasRShGS$	---	$RasGTP$	$RShGS$	$\kappa_2(K_{RasRShGS}\cdot q_{RasRShGS} - K_{RasGTP}\cdot q_{RasGTP}\cdot K_{RShGS}\cdot q_{RShGS})$
**3**	$RasGTP$	GAP	$RasGAP$	---	$\kappa_3(K_{RasGTP}\cdot q_{RasGTP}\cdot K_{GAP}\cdot q_{GAP} - K_{RasGAP}\cdot q_{RasGAP})$
**4**	$RasGAP$	---	GAP	$RasGDP$	$\kappa_4(K_{RasGAP}\cdot q_{RasGAP} - K_{GAP}\cdot q_{GAP}\cdot K_{RasGDP}\cdot q_{RasGDP})$
**5**	$RasGTP$	---	Ras	---	$\kappa_5(K_{RasGTP}\cdot q_{RasGTP} - K_{Ras}\cdot q_{Ras})$
