# Supplementary material for: A semantics, energy-based approach to automate biomodel composition
Source: PLoS One. 2022 Jun 3;17(6):e0269497. doi: 10.1371/journal.pone.0269497 (PMC9165793; doi:10.1371/journal.pone.0269497)
Supplement: S5 Table — (PDF) [file pone.0269497.s007.pdf]

S5 Table. Kinetic and bond graph parameters of the species in the Ras activation intermediate pathway model.

Species	Brightman & Fell	Bond graph version	
	q (dimensionless)	K (nM^{-1})	q (nM)
$\boldsymbol{RShGS}$	0	0.13	0
$\boldsymbol{RasGDP}$	19800	0.13	1.98
$\boldsymbol{RasRShGS}$	0	10.47	0
$\boldsymbol{RasGTP}$	200	0.0064	2e-2
$\boldsymbol{GAP}$	15000	0.553	1.5
$\boldsymbol{RasGAP}$	0	42.72	0
$\boldsymbol{Ras}$	0	16.07	0
