# Supplementary material for: A semantics, energy-based approach to automate biomodel composition
Source: PLoS One. 2022 Jun 3;17(6):e0269497. doi: 10.1371/journal.pone.0269497 (PMC9165793; doi:10.1371/journal.pone.0269497)
Supplement: S6 Table — (PDF) [file pone.0269497.s008.pdf]

S6 Table. Kinetic and bond graph parameters of the reactions in the Ras activation intermediate pathway model.

Reactions	Brightman & Fell		Bond graph version
	k^{+}	k^{-}	κ (nmol.s[-1])
$\boldsymbol{Re}:\boldsymbol{\kappa 1}$	0.0163	10	0.95
$\boldsymbol{Re}:\boldsymbol{\kappa 2}$	15	---	1.43
$\boldsymbol{Re}:\boldsymbol{\kappa 3}$	0.005	60	1.4
$\boldsymbol{Re}:\boldsymbol{\kappa 4}$	720	---	16.85
$\boldsymbol{Re}:\boldsymbol{\kappa 5}$	0.0012	3	0.186
