# Supplementary material for: A semantics, energy-based approach to automate biomodel composition
Source: PLoS One. 2022 Jun 3;17(6):e0269497. doi: 10.1371/journal.pone.0269497 (PMC9165793; doi:10.1371/journal.pone.0269497)
Supplement: S1 Text — Appendix A: Connectivity matrix example. B: Parameter estimation for step 4 in the EGFR pathway model. Appendix C: An example of composing two reactions in bond graphs. Fig A: An example network with its connectivity matrix. Fig B: The irreversible Michaelis-Menten and its equivalent approximated reversible mass action kinetics for step 4 in the EGFR signalling pathway model. (PDF) [file pone.0269497.s009.pdf]

A Connectivity matrix example

The connectivity matrix (CM) for a system with m elements can be described as $CM \in \{0,1\}_{mxm}$, where:

$$CM_{ij} = \begin{cases} 1, & if\ a_i and\ a_j are\ connected \\ 0, & otherwise. \end{cases} \tag{1}$$

Fig A shows the connectivity matrix for a sample network. The connectivity matrix for bond graph models is symmetric ($CM_{ij} = CM_{ji}$), representing the bidirectional flow of energy between the bond graph components.

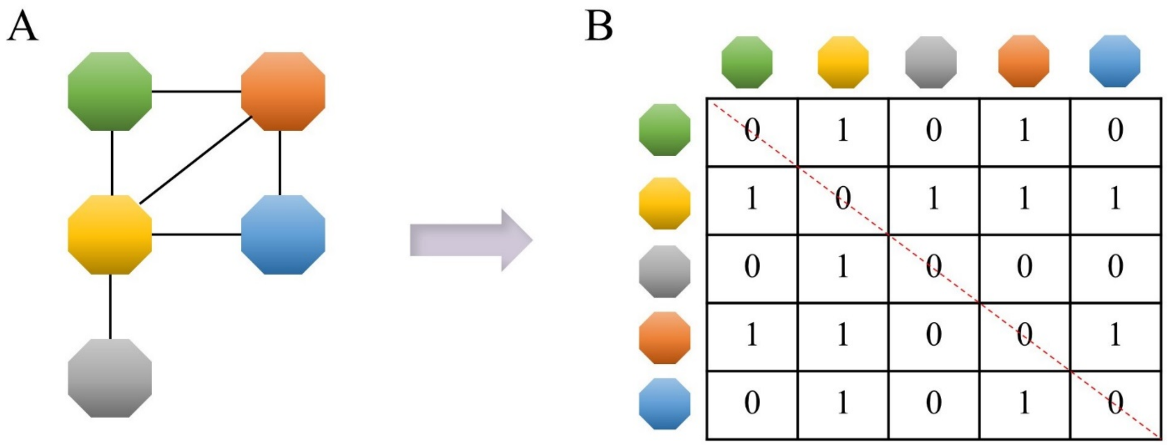

Figure A An example network with its connectivity matrix. (A) The network topology of the connections between the elements of a system; (B) The connectivity matrix of the system. 0 means no connection and 1 means connection. The dashed red line shows the diagonal of the matrix to demonstrate its symmetry.

B Parameter estimation for step 4 in the EGFR pathway model

The original reaction in step 4 is described using the Michaelis-Menten kinetics as:

$$v_4 = V_{max4} \frac{q_{RP}}{K_{m4}+q_{RP}} \tag{2}$$

Where V_{max4} and K_{m4} are the Michaelis-Menten constants and q_{RP} is the reactant (RP) concentration. We require to approximate the irreversible Michaelis-Menten kinetics with the reversible mass-action kinetics as follows:

$$v_4 = \ \kappa_4 (K_{RP}\, q_{RP} - K_{R2}\, q_{R2}\, K_{Pi}\, q_{Pi}) \tag{3}$$

Where κ_4 is the reaction rate constant in step 4, K_{RP}, K_{R2}, and K_{Pi} are the thermodynamic constants for RP, $R2$, and Pi, and q_{R2}, q_{Pi} are the concentrations for $R2$ and Pi, respectively. We have added Pi to the products to account for the missing source of potential.

Since the thermodynamic constants for the reactants and products have been calculated in the previous steps and the species concentrations are known from the simulation data of the original model, we only need to find κ_4. We used scipy.optimize.curve_fit to fit the simulation data to Eq (3) using the least square error with the initial conditions from the original CellML files. Fig B illustrates the reaction rate in step 4 for the irreversible Michaelis-Menten and the approximated reversible mass-action kinetics.

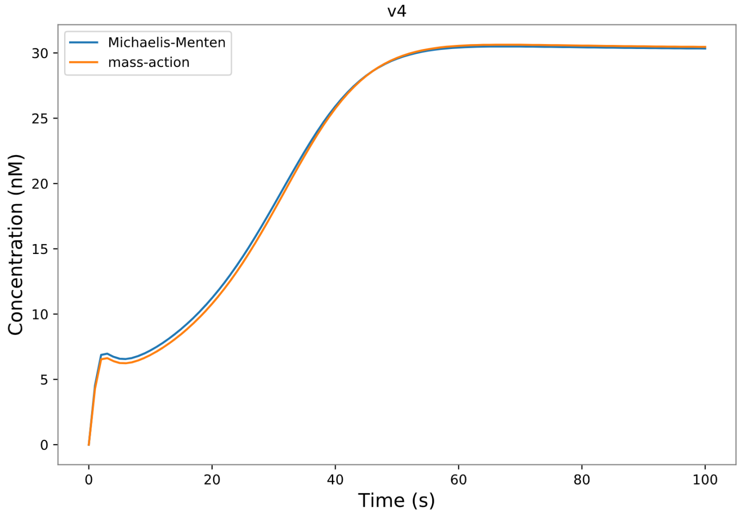

Figure B: the irreversible Michaelis-Menten and its equivalent approximated reversible mass-action kinetics for step 4 in the EGFR signalling pathway model.

C An example of composing two reactions in bond graphs

Here, we demonstrate the composition of two reactions in bond graphs. Reaction 1 is defined as $\alpha A + B \rightleftharpoons C$ and Reaction 2 is defined as $C \rightleftharpoons D$.

In bond graphs, components connected to a $'1\!:v'$ junction share the same flow and the sum of efforts (chemical potential in biochemistry) equals the inward effort. On the other hand, components connected to a $'0\!:u'$ junction share the same effort and the sum of flows equals the outward flow. All the reactants on the left hand side of a reaction are constrained to the same molar flow rate defined by the reaction and hence, they are all connected to the reaction via a $'1\!:v'$ junction. Same thing goes for the products on the right hand side of the reaction too. If there is only one species at either side of a reaction, since it is not sharing the flow with any other species, it can be directly connected to the reaction (without attaching to a $'1\!:v'$ junction). The stoichiometric coefficient expressed as $TF\!:\alpha$ in Reaction 1, affects the rate of consumption of A by a factor of α. The conservation equations for the species in Reaction 1 are as follows:

$$v_A = \frac{dq_A}{dt} = -\alpha v_1 \tag{4}$$

$$v_B = \frac{dq_B}{dt} = -v_1 \tag{5}$$

$$v_C = \frac{dq_C}{dt} = v_1 \tag{6}$$

and the constitutive relation for Reaction 1 is:

$$v_1 = \kappa_1((K_A q_A)^\alpha K_B q_B - K_C q_C) \tag{7}$$

Similarly, the conservation equations for the species in Reaction 2 are as follows:

$$v_C = \frac{dq_C}{dt} = -v_2 \tag{8}$$

$$v_D = \frac{dq_D}{dt} = v_2 \tag{9}$$

and the constitutive relation for Reaction 2 is:

$$v_2 = \kappa_2(K_C q_C - K_D q_D) \tag{10}$$

The molar flow rate of each of the species is determined by solving the conservation equations in each reaction.

Since the species C participates in both reactions, Reactions 1 and 2 can be integrated into one composed reaction through merging the C components. Due to the possibility of appending and deleting components in bond graphs, one of the C components will be removed during the composition and the other one will be kept. Then their $'0{:}\,u'$ junctions will be connected by adding a bond between them. In bond graphs the connection between similar junction types ($'0{:}\,u'$ or $'1{:}\,v'$) can be reduced to only one junction since physically they are carrying the same potential and flow variables. The conservation equations for A, B, and D in the composed reaction remain the same as in Eqs (4), (5), and (9) but it differs for C:

$$v_C = \frac{dq_C}{dt} = v_1 - v_2 \tag{11}$$

The constitutive relations for Reaction 1 and 2 remain the same as in Eqs (7) and (10).

In conclusion, when a species is shared and merged between two reactions, the conservation law at its corresponding $'0{:}\,u'$ junction imposes a change in equations to include other flows added by the new emerged bonds. In this case, C was produced in Reaction 1 and consumed in Reaction 2 independently but when merged, C was both produced and consumed at the same time, implying that both reaction rates (v_1 and v_2) must be considered in its conservation equation.
