# Supplementary material for: A semantics, energy-based approach to automate biomodel composition
Source: PLoS One. 2022 Jun 3;17(6):e0269497. doi: 10.1371/journal.pone.0269497 (PMC9165793; doi:10.1371/journal.pone.0269497)
Supplement: Attachment — Submitted filename: REVIEW.pdf [file pone.0269497.s010.pdf]

I am of the opinion that energy-based model composition will play a critical role in expanding the utility of reaction models in biochemistry. The authors have applied multiple innovative strategies such as bond graphs, templating, automated semantic inference, and automated merging to their model composition pipeline, so the premise of the paper is sound. However, it disappoints me to **recommend rejecting this manuscript** on several counts. **Issue 3a and 3f below are critical reasons** behind recommending rejection over revision.

1. It fails to properly introduce the problems being solved, how they originate in biology, and how the proposed methods solve those problems.
2. It fails to introduce the technical background of the methods being deployed in a manner accessible to a general biological audience, or even an audience interested in biochemical models.
3. It has multiple issues with the demonstrated results: questionable choices in merging, insufficient demonstration of proof of concept, poor structuring and comparison of simulation results.
4. Severe readability and copy-editing issues with the text.

Issue 1: Introducing the problems being solved

The introduction takes for granted that the reader is aware of scale issues in biochemical modeling. First, they must motivate why system-level modeling of biochemical reactions is difficult. This includes providing information on

a) how biochemical complexity affects species semantics, and how that creates problems for model composition,
b) detailed balance and thermodynamic consistency concepts, how they apply to biology, and how current approaches fail at this,
c) how energy-based composition resolves the issue of thermodynamic consistency.

The manuscript touches upon these points briefly in several places (e.g., white-box approach, etc.). But it never provides a cohesive structured argument accessible to the reader. It is not sufficient to simply state that other methods produce infeasible models, but to demonstrate what infeasibility means in this context and why it happens.

Issue 2: Introducing the technical background

Both in the introduction and in the methods section, the authors over-explain the *mechanics* of what they do, but under-explain the *concepts* relevant to understanding. Thus, it feels like reading a tutorial without grasping the scientific intuition behind it.

a) A more detailed representation of related work is necessary. E.g., what are the issues with SBML-hierarchical and state of the art in model composition? What are post-composition adjustments?
b) For bond graphs, the authors need to explain separately bond graph theory, bond graph terms and what they mean, and instructions on how to build/visualize/understand bond graphs. Combining all of these into a single section (Sec 2.2) makes it hard to understand. For example, how does one decide where to glue edges on the bond graph? In Fig 3A, 0:u species nodes are connected to a 1:v reaction node, whereas in Fig 5, 0:u nodes are often connected directly to Re:k nodes. My guess is that it does not matter because of some underlying bond graph theory, but I should not have to resort to guesswork in the face of insufficient explanation.

a) Explain the connection between thermodynamic terms and kinetic terms, e.g., is the thermodynamic constant of a species the same as the more familiar free energy of formation of species? What is a dissipative process?
b) Explain the connection between detailed balance and energy conservation. What does it mean to obey physical laws in the context of reactions (it is not sufficient to just state that they should be obeyed)? The manuscript does not explain it to the general audience: specifically, that on a per-mol basis, energy should be conserved around a loop of reactions, which places constraints on the relationships between kinetic parameters.
c) Explain to a general audience how energy-based composition automatically produces thermodynamically consistent models: specifically that reframing kinetic parameters using energies of formation of species leads to conservation laws preserved around loops.
d) Bond graph model simulation is not mentioned at all. However, results from the simulation are shown. How is bond graph model simulation different from or related to known methods like ODE integration and Gillespie SSA? This is something a general modeling audience will be completely unaware of.
e) For semantics-based composition, the methods section has too many special terms that are not fully defined. Sufficient background needs to be provided on annotations and ontologies and a simple example must be used to demonstrate how merging occurs.
f) For the composition section, use a small bulleted list to convey the elements of the pipeline. Then explain each element in detail.

Issue 3: Problems with Results

a) The EGFR-Ras-MAPK example shown is small enough to be in a tutorial, **but it is not sufficient to be a full demonstration of the proof of concept**. At the very least, attempt must be made to merge multiple (> 2) models hierarchically.
b) I'm not very happy with the decision to merge Sos species with Ras. It could've been easily avoided by using a third model with two simple Sos-Ras reactions. In fact, this highlights a potential problem with the hierarchical composition approach: what happens when you start to merge two models, but then you identify missing elements missing that require additional modeling? I'm assuming this comes under post-composition adjustments.
c) The interplay between manual selection (e.g., indicating Sos as Ras) vs automated semantic inference (e.g., inferring Sos to be a specific biochemical entity) should be clearly delineated and its effects discussed.
d) The figures for the results are poorly structured. Whenever figure panels are being compared in the text, they should be juxtaposed in the same figure. For example, it would be useful to place MAPK cascade simulation results and MAPK bond graph simulation results in the same panel for direct comparison and verification.
e) In the section examining effect of ATP concentrations, the inputs provided are not mentioned (e.g., what Ras concentration is used for each curve in Fig 15). In fact, comparing the curves at a single parameter point is not sufficient to make a general statement.
f) A critical shortcoming of the manuscript is that **it does not even examine the composed model in detail**. The goal of model composition is to enable the pieces of one model to influence the effects of another model. In this case, the goal of merging EGFR model with MAPK is to examine the effect of EGF concentrations on MAPK. What new types of analysis are now possible on your merged model

that you couldn't do with the unmerged models? What predictions does it do that confirm or contradict existing experiments or predictions from the many EGFR-MAPK models in the literature?

Suggestions on related work

The innovation of this work is in the application of bond graphs to reaction composition in biochemical systems. However, it is being increasingly considered that the species complexity of biochemical cells will limit our ability to compose large models from small ones due to inconsistencies in species semantics across models (touched upon in this manuscript). The authors are encouraged to check out rule-based modeling, where species semantics are formally embedded in graph structures and the energy-based extension of rule-based modeling, which largely applies the same thermodynamic principles used in this manuscript and produces consistent models that obey detailed balance. References:

Rule-based modeling:

- Chylek et al. Physical Biology 2015
- Harris et al. Bioinformatics 2016
- Boutillier et al. Bioinformatics 2018

Energy-based rule-based modeling

- Ollivier et al PLoS Comp Bio 2010
- Sekar et al IEEE BIBM 2016
- Justin Hogg Ph.D. dissertation Chapter 2, University of Pittsburgh, 2013
- Thermodynamic Graph Rewriting, Danos et al. arxiv 2015

Issue 4: Problems with Readability

Part of scientific communication is to emphasize clarity and directness. As it stands, the text is too verbose and unstructured and is not fully copy-edited. Some suggestions to make it readable:

a) Using passive voice unnecessarily makes sentences long and complicated. E.g., Instead of saying "modifying a network is easily performed by…", you can say "to modify a network, one can…" It also makes things difficult to understand as to whether it was done automatically or manually, particularly in several places in the methods section.
b) Some words are overused and do not convey any meaning to the reader. For example, I fail to understand what is "generic" about the composition pipeline.
c) Some sentences are unnecessarily long without providing any additional meaning. E.g., instead of saying "we employed the idea of having symbolic bond graph templates", you can simply say "we built symbolic bond graph templates".
d) Figure captions need to provide sufficient information so that they can be read in isolation. This means the caption should briefly summarize how the figure is referenced in the paper. E.g. Fig 15 caption does not even mention which model is used.
e) The text in figures is extremely tiny relative to the size of the figure and unreadable. Effort should be made so that the figure looks good printed on paper.
f) Many paragraphs begin with extra-long sentences that run on. E.g. lines 48-50 packs too many different concepts into a single sentence. This is unnecessary and can be broken down.

g) Sections should begin with a brief paragraph summarizing the section. Each paragraph should have a first sentence summarizing the paragraph.
h) In many places, special terms are used before being defined, which is poor form. For example, physical feasibility in line 20 is defined only in line 26. Similarly, symbolic models in line 96 is used first and then explained. Semantics-based in line 40 has no explanation. Using "… will be explained later" is also poor form and shows lack of narrative.
