# Supplementary material for: A semantics, energy-based approach to automate biomodel composition
Source: PLoS One. 2022 Jun 3;17(6):e0269497. doi: 10.1371/journal.pone.0269497 (PMC9165793; doi:10.1371/journal.pone.0269497)
Supplement: Attachment — Submitted filename: Response to Reviewers.pdf [file pone.0269497.s012.pdf]

Response to Reviewers

A semantics, energy-based approach to automate biomodel composition

We thank the reviewers for taking the time to read our revised manuscript. We have included the latest suggestions from Reviewer#2 and Reviewer#3 as detailed below. The reviewer comments will be shown in black, our responses in green and quotations from the revised manuscript in blue.

Reviewer #2:

The new version of the text is much improved and adequately addresses my major concerns. The github repo looks good too, but could perhaps benefit from a link to BondGraphTools (or a mention that it's pip installable).

We have added a link to BondGraphTools installation steps on our GitHub repository (Readme file).

Reviewer #3:

Dear authors,

Thank you for answering my comments and for the modifications you made. You addressed all the questions I raised, but some of your points could still be explained more clearly. I won't insist on any one of them. I'm listing them below for your information and leave it to you to decide which changes you would like to make.

Sincerely,
Your reviewer #3

(38-40) .... an energetic and multi-physics framework that explicitly models energy to ensure adherence to the laws of physics and is executable in multi-physics modelling."

-> For me, the term "multi-physics framework" is still a bit vague, you could briefly explain what you mean by it in this specific context; also "energetic" and "models energy" will not be clear to some readers, because "energy", without further specification, can mean many things.

We have added an example of "multi-physics" systems and "energetic modelling" in the following text.

(39-42) ... an energetic and multi-physics framework that explicitly models energy (expressing kinetic rate laws of biochemical reactions in terms of chemical energy level differences [17]) and ensures adherence to the laws of physics and is executable in multi-physics modelling (such as cardiomyocytes electromechanical coupling).

(116-118) ... provides a reliable and consistent framework that first tracks energy transfer; secondly ensures that reactions can only operate in the direction of decreasing chemical potential; ...

-> Again, "energy transfer" is not clear and should be explained.

We have modified the following text for clarity.

(119-121) ... provides a reliable and consistent framework that is consistent with energy conservation; secondly ensures that reactions can only operate in the direction of decreasing chemical potential; ...

Section 2.2 explains the usage of bond graph modeling of biochemical reactions, but it remains unclear how parameters, rate law formulae, and other data attached to the nodes will be treated during model composition. Is an enzymatic rate law a property attached to the reaction node?
We believe this has been addressed in the comment on line 109.

-> I don't see how this is addressed in the comment on line 109, maybe you can explain this more explicitly.

We have demonstrated this with an example given in Fig 2 (showing how merging in bond graphs occurs graphically) and how the rate law formulae and conservation laws change during model composition in S1 Text.C.
Yes, the enzymatic rate law is a property attached to the reaction node but instead of relating the reaction fluxes to concentrations, bond graphs relate fluxes to chemical potentials. We have discussed this in lines 40-41 of the manuscript.

-> The font size in Figure 8 is now ok, but the font size in figure 6C could still be increased

We have increased the font size in Figure 6.C.

583: “Eventually, the generated mathematical equations in the bond graph environment can be converted into CellML for simulation and reproducibility.” Would the results models again have the form of a “normal model”, or do they still look very “bond-graph like”, e.g. with non-biological components representing junctions? (And I have the same question for a (potential, future) conversion to SBML models).
We have explained the form of the converted bond graph model in CellML in the following text.
(822-825) The regenerated bond graph model encoded in CellML will lose its graphical structure and the model will be expressed in a system of ODEs. Since we can convert the exported bond graph ODEs into MathML format, the biochemical equations would be also expressible in SBML.

-> Thank you for the clarification. I can see that it is expressible in SBML; but does it still have the "natural" structure of an SBML model, with concentrations changes described by stoichiometric coefficients and reaction fluxes (described in "reaction" elements), or will it just be a collection of ODEs? Please clarify.

We believe that the conversion from bond graph models of biochemical systems to the natural structure of SBML models is possible due to the following reasons:

1. The constitutive equations for **Re components** (reactions) in bond graphs are expressed in terms of chemical potential (energy) differences and hence automatically account for energy conservation. These equations can be directly used as kinetic law expressions in SBML reactions too. There is no need to change the derived mathematical equations from bond graphs and they can be directly applied to SBML “reaction” elements.
2. The **Ce components** and **TF transformers** in bond graphs represent the “species” and “stoichiometry” in SBML models, respectively. Therefore, the transfer of these bond graph elements’ specific parameters to their SBML corresponding elements is conceivable.
3. As well as the reactant(s)-reaction-product(s) are extractable from SBML models, same

relationships can be deduced from bond graph models of biochemical systems and inserted in SBML models.

We have modified the following text to include a summary of the above-mentioned ideas.

(825-830) The regenerated bond graph model encoded in CellML will lose its graphical structure and the model will be expressed as a system of ODEs. Since we can convert the exported bond graph ODEs into MathML format, the biochemical equations would be also expressible in SBML. The structure of such SBML models will be preserved since the required parameters, rate laws, and reactant(s)-reaction-product(s) relationships are extractable from the generated bond graph model.

(782-791) Here, we have selected models encoded in CellML because CellML can deal with models that are note purely biochemical,

-> typo "note"

We thank the reviewer for pointing out the typo and have corrected it.

(31-34) Several formulations and frameworks have been developed to ensure biochemical models follow the laws of thermodynamics ([15–17]) but most of them are purely mathematical and are difficult to implement for model composition.

-> I don't think they would be difficult to implement for model composition (at least, if some standardised rate laws are used), I think the main point here is that they HAVEN'T been implemented for model composition.

We have included the reviewer's comment in the following text to make it clearer.

(31-35) Several formulations and frameworks have been developed to ensure biochemical models follow the laws of thermodynamics ([15–17]) but most of them are purely mathematical and are often difficult to implement for model composition due to non-standardised rate laws, and lacking an easy append/delete graphical structure.
